# Supplementary material for: Quantification of Enhydrin and Uvedalin in the Ethanolic Extract of Smallanthus sonchifolius Leaves Validated by the HPLC Method
Source: Molecules. 2023 Feb 17;28(4):1913. doi: 10.3390/molecules28041913 (PMC9959401; doi:10.3390/molecules28041913)
Supplement: Supplementary file 1 [file molecules-28-01913-s001.zip › molecules-2137573-supplementary.pdf]

SUPLEMENTARY

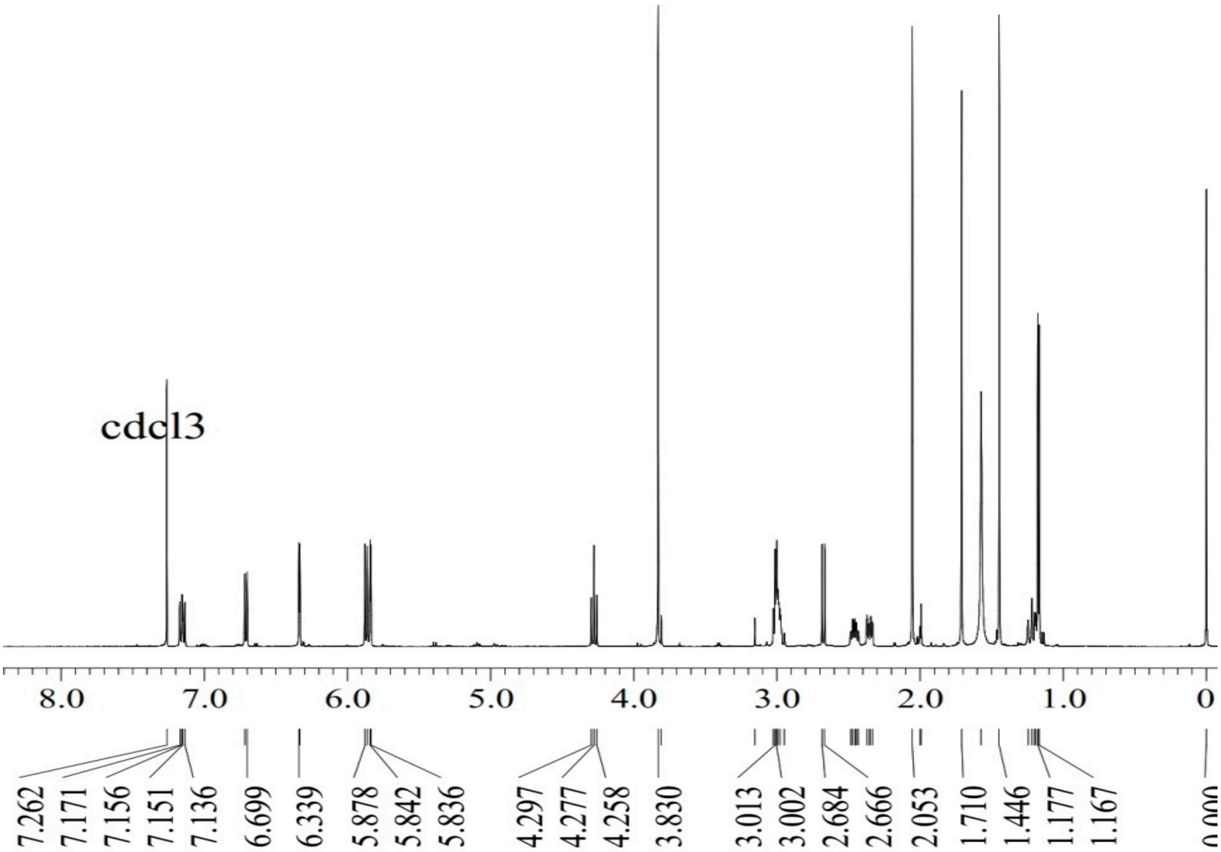

Figure S1. ^{1}H-NMR Spectra of (**1**) (500 MHz in $CDCl_3$)

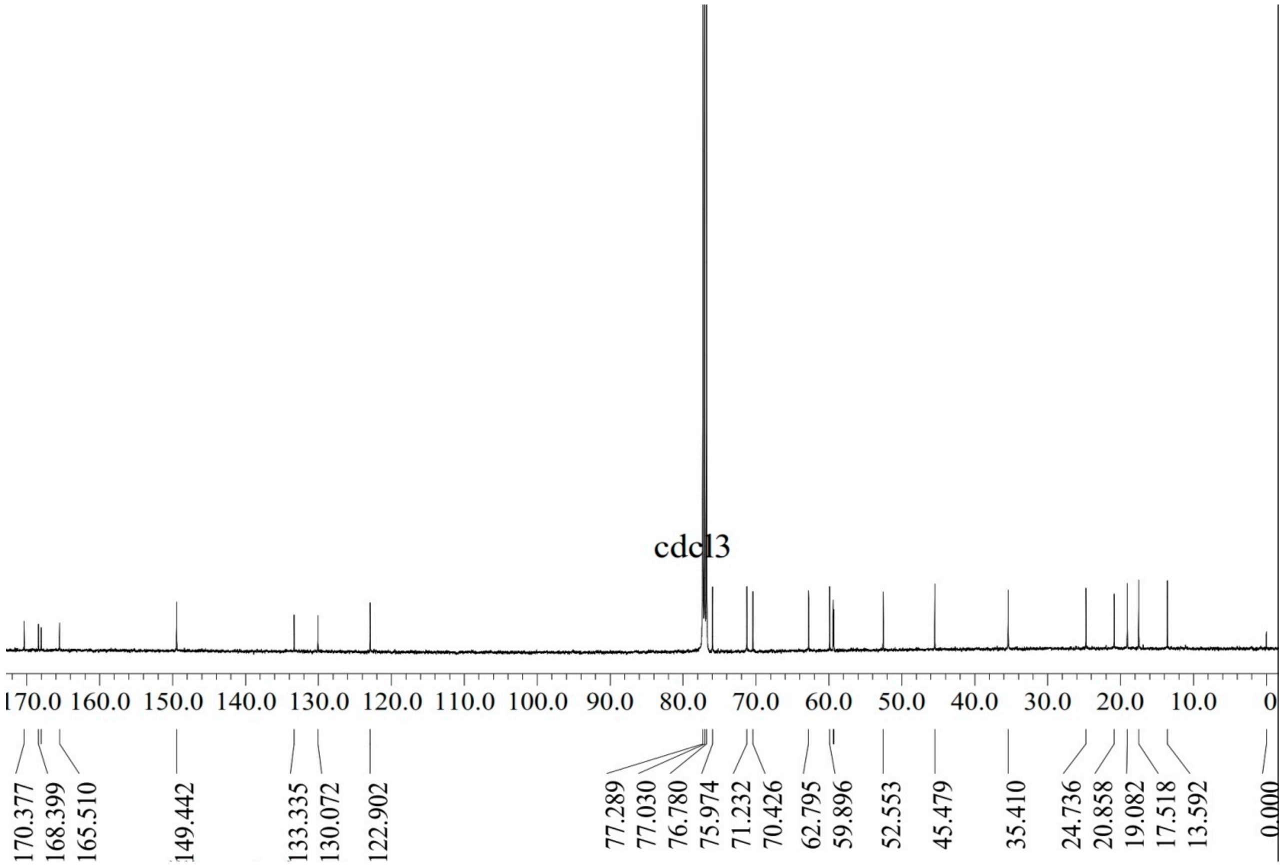

Figure S2. ^{13}C-NMR Spectra of (**1**) (500 MHz in $CDCl_3$)

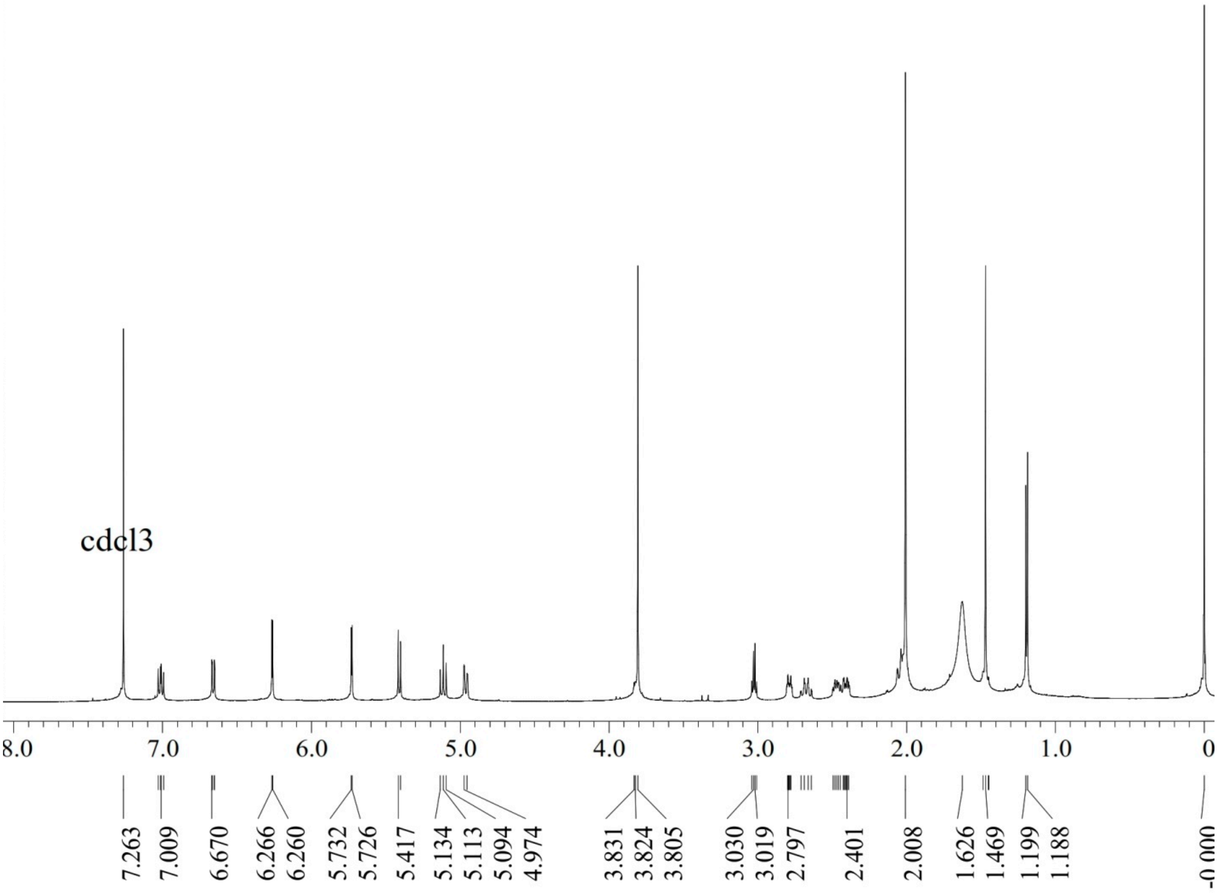

Figure S3. ^{1}H-NMR Spectra of (**2**) (500 MHz in $CDCl_3$)

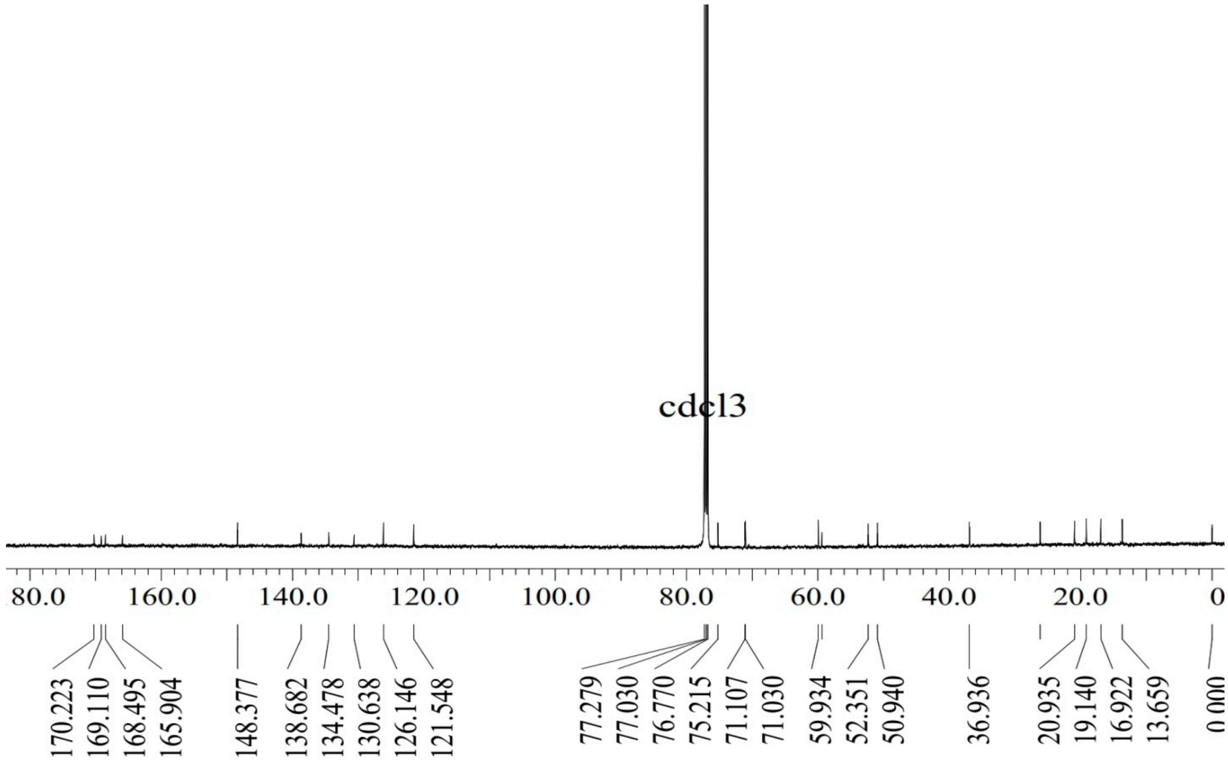

Figure S4. ^{13}C-NMR Spectra of (**2**) (500 MHz in $CDCl_3$)

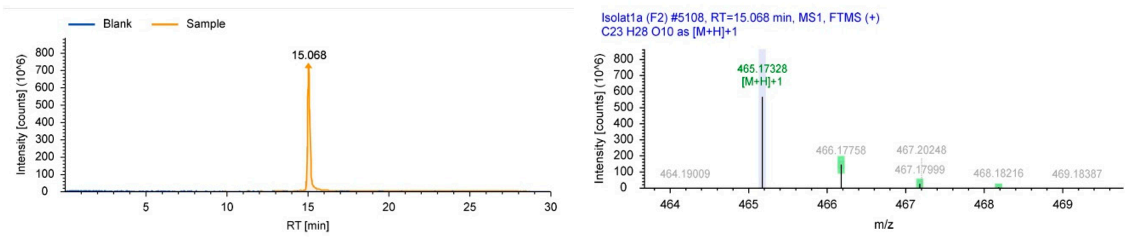

Figure S5. LC-MS Spectra of (**1**)

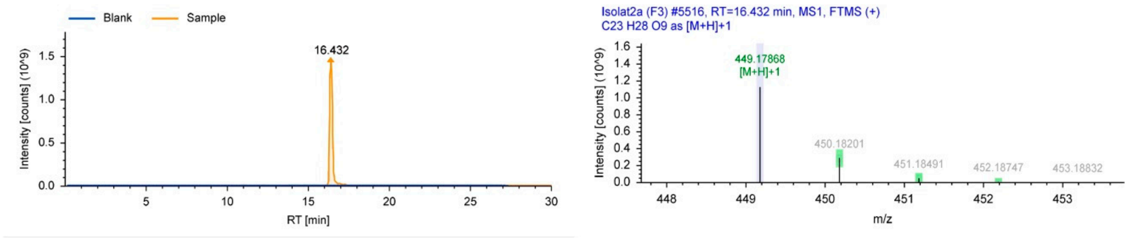

Figure S6. LC-MS Spectra of (**2**)
